# Mononeuritis multiplex following immune checkpoint inhibitors in malignant pleural mesothelioma

**DOI:** 10.3389/fneur.2024.1338899

**Published:** 2024-01-25

**Authors:** Antonio Farina, Manon Escalere, Matthias Dion, Martin Moussy, Antoine Pegat, Macarena Villagrán-García, Perrine Devic, Anaïde Lamiral, Antoine Seyve, Karine Aure, Adrien Wang, Lucas Gorza, Nathalie Streichenberger, Thierry Maisonobe, Jerome Honnorat, Cristina Birzu, Dimitri Psimaras, David Weisenburger-Lile, Bastien Joubert

**Affiliations:** ^1^Centre de Référence Français des Syndromes Neurologiques Paranéoplasiques et des Encéphalites Auto-immunes, Hospices Civils de Lyon, Hôpital Neurologique, Bron, France; ^2^MeLiS—UCBL-CNRS UMR 5284—INSERM U1314, Université Claude Bernard Lyon 1, Lyon, France; ^3^Unité de Neurologie et de Neurovasculaire, Foch Hospital, Suresnes, France; ^4^Service de Neurologie, Centre Hospitalier Lyon Sud, Hospices Civils de Lyon, Lyon, France; ^5^Service ENMG et Pathologies Neuromusculaires, Hôpital Neurologique Pierre Wertheimer, Hospices Civils de Lyon, Lyon, France; ^6^Service de Neuro-Oncologie, Hôpital Neurologique, Hospices Civils de Lyon, Lyon, France; ^7^Service de Neuropathologie, Groupement Hospitalier Est, Hospices Civils de Lyon, Lyon, France; ^8^Université Claude Bernard Lyon 1, Institut NeuroMyogène, CNRS UMR 5261—INSERM U1315, Lyon, France; ^9^Département de Neurophysiologie Clinique, AP-HP, Pitié-Salpêtrière Hospital, Paris, France; ^10^Sorbonne Université, Publique-Hôpitaux de Paris, Groupe Hospitalier Pitié-Salpêtrière-Charles Foix, Paris, France; ^11^INSERM, CNRS, Assistance Publique-Hôpitaux de Paris, Institut du Cerveau Et de La Moelle Épinière, Sorbonne Université, Paris, France

**Keywords:** vasculitic neuropathy, nerve vasculitis, mononeuritis multiplex, immune checkpoint inhibitor, neurological toxicity, neurological immune-related adverse events, n-irAEs

## Abstract

**Introduction:**

Mononeuritis multiplex is frequently related to vasculitic neuropathy and has been reported only sporadically as an adverse event of immune checkpoint inhibitors.

**Methods:**

Case series of three patients with mononeuritis multiplex—all with mesothelioma—identified in the databases of two French clinical networks (French Reference Center for Paraneoplastic Neurological Syndromes, Lyon; OncoNeuroTox, Paris; January 2015–October 2022) set up to collect and investigate n-irAEs on a nationwide level.

**Results:**

Three patients (male; median age 86 years; range 72–88 years) had pleural mesothelioma and received 10, 4, and 6 cycles, respectively, of first-line nivolumab plus ipilimumab combined therapy. In patient 1, the neurological symptoms involved the median nerves, and in the other two patients, there was a more diffuse distribution; the symptoms were severe (common terminology criteria for adverse events, CTCAE grade 3) in all patients. Nerve conduction studies indicated mononeuritis multiplex in all patients. Peripheral nerve biopsy demonstrated necrotizing vasculitis in patients 1 and 3 and marked IgA deposition without inflammatory lesions in patient 2. Immune checkpoint inhibitors were permanently withdrawn, and corticosteroids were administered to all patients, leading to complete symptom regression (CTCAE grade 0, patient 2) or partial improvement (CTCAE grade 2, patients 1 and 3). During steroid tapering, patient 1 experienced symptom recurrence and spreading to other nerve territories (CTCAE grade 3); he improved 3 months after rituximab and cyclophosphamide administration.

**Discussion:**

We report the occurrence of mononeuritis multiplex, a very rare adverse event of immune checkpoint inhibitors, in the three patients with mesothelioma. Clinicians must be aware of this severe, yet treatable adverse event.

## 1 Introduction

Immune checkpoint inhibitors (ICIs) are monoclonal antibodies that target crucial negative regulators of the immune system, mainly cytotoxic T-lymphocyte-associated protein 4 (CTLA-4), programmed cell death protein 1 (PD-1), and PD-1-ligand (PDL-1), leading to enhanced anti-tumor immune responses, but also triggering toxicities that may affect any organ and are defined immune-related adverse events (1). Neurological immune-related adverse events (n-irAEs) are rare (1–3% of ICIs-treated patients) and heterogeneous in terms of neurological structures affected, response to corticosteroids, and outcomes (1–3). Mononeuritis multiplex, a neuromuscular disorder frequently related to systemic vasculitis, was reported only sporadically as a n-irAE (3). Herein, we report three mesothelioma patients with mononeuritis multiplex, including two with pathology-proven vasculitis, following a recently approved combined anti-PD-1 (nivolumab)/anti-CTLA-4 (ipilimumab) first-line treatment (4).

## 2 Methods

The study is a case series of three previously unpublished patients with mononeuritis multiplex—all with mesothelioma—identified in the databases of two French clinical networks (French Reference Center for Paraneoplastic Neurological Syndromes, Lyon; OncoNeuroTox, Paris; January 2015–October 2022) set up to collect and investigate Common Terminology Criteria for Adverse Events (CTCAE) grade ≥ 2 n-irAEs on a nationwide level. Data were collected retrospectively from all the available medical charts. Disease severity was classified according to the CTCAE v5.0 (1).

### 2.1 Ethical considerations

Approval for this study was granted by the institutional review board of the *Université Claude Bernard Lyon 1* and *Hospices Civils de Lyon* (69HCL21-474), and the study was registered to the *Commission nationale de l’informatique et des libertés* (CNIL, 21-5474). Patients’ informed consent was obtained according to the Declaration of Helsinki and its later amendments.

## 3 Results

**Figure 1:**
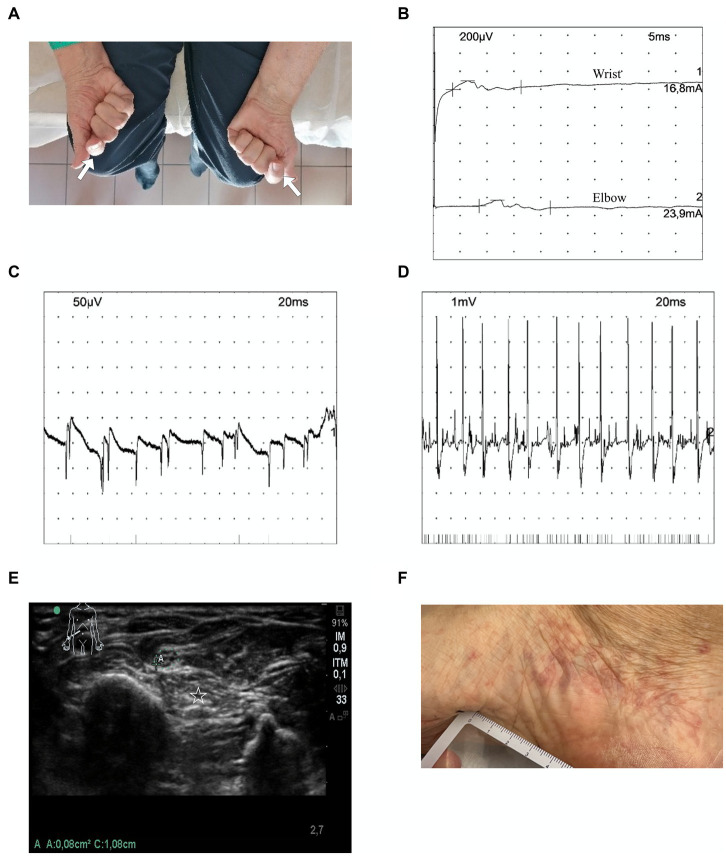
Clinical, ultrasonography, and electrophysiological findings in patient 1. **(A)** The patient was asked to clench his fists. Note the impaired thumb opposition and index flexion on both sides (arrows). **(B)** Nerve conduction study (day 28 from clinical onset) of the left median nerve on musculus abductor pollicis brevis, with distal stimulation at the wrist, and proximal stimulation at the elbow (200 μV/div, 5 ms/div): very low distal amplitude (0.1 mV), with normal distal latency (3.6 ms) and normal motor conduction velocity at 53.1 m/s. On the right side, the muscle was unexcitable even by the maximum output of nerve stimulation. **(C)** Myography of left muscle flexor carpi radialis (day 28 from clinical onset) at rest: positive sharp waves (++) in the muscle (50 μV/div, 20 ms/div). **(D)** Myography of left muscle flexor carpi radialis (day 28 from clinical onset) at effort (1 mV/div, 20 ms/div): severely impaired motor unit recruitment, while a motor unit is firing at 30 Hz. **(E)** Ultrasonography: normal cross-sectional area (0.08 cm^2^) of the right median nerve (*N* < 0.10 cm^2^), at forearm (1/3 distal; letter A in white, with a green circle for the epinerve). Hyperechogenicity of right flexor digitorum profundus suggesting denervation (star). **(F)** Purpuric lesions of the medial aspect of the right foot. μV, microvolt; mV, millivolt; ms, milisecond, m/s, meter/second; Div, division; and Hz, Hertz.

**Figure 2:**
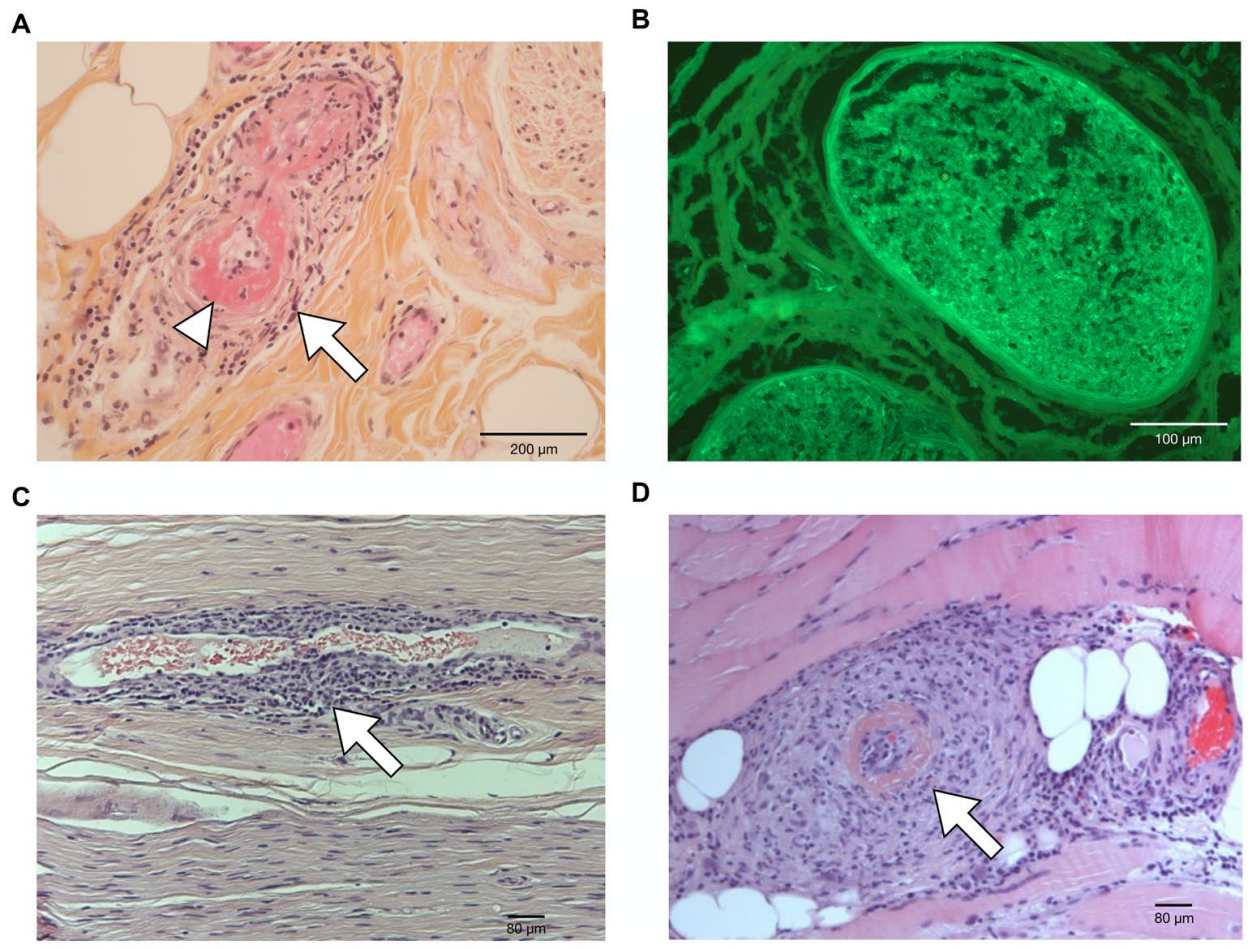
Histological findings. **(A)** Sural nerve biopsy (hematoxylin–phloxine–saffron) in patient 1 showing endocapillary mononuclear inflammatory infiltrates (arrow) with fibrinoid necrosis (arrowhead). **(B)** Sural nerve biopsy (indirect immunofluorescence) in patient 2 demonstrating marked endoneurial IgA deposition (green fluorescence). **(C)** Superficial fibular nerve biopsy in patient 3 showing mononuclear inflammatory infiltrate surrounding one endoneural small vessel (arrow). **(D)** Fibularis brevis muscle biopsy in patient 3 showing a mononuclear inflammatory infiltrate associated with thrombosis and fibrinoid necrosis of a medium caliber vessel (arrow).

Three patients (male; median age 86 years; range 72–88 years) had been diagnosed with pleural mesothelioma and had received 4–10 cycles of first-line nivolumab plus ipilimumab at the time of neurological symptoms, which consisted of left-hand painful paresthesia and weakness spreading 1 week later to the right hand (patient 1), four-limb burning pain, lower limb weakness and gait instability (patient 2), and left drop foot and numbness, followed by bilateral (left>right side) hand weakness and numbness (patient 3). Neurological examination showed weakness and sensory loss and/or allodynia of individual peripheral nerves in patient 1 (bilateral median, Figure 1A) and patient 3 (left peroneal, left radial, and bilateral ulnar), lower limb weakness with a diffuse distribution in patient 2, and sensory ataxia in patients 2 and 3 (Table 1). In addition, patient 1 had purpuric lesions on his hands and feet (Figure 1F), while patient 2 had steroid-responsive immune-related arthritis of the scapular and pelvic girdles, which appeared shortly before the onset of neurological symptoms. Clinical presentation was severe in all patients (CTCAE grade 3). Nerve conduction studies indicated asymmetric axonal sensory and motor mononeuritis multiplex with active denervation in the three patients (Table 1; Figures 1B–D). Nerve and muscle ultrasonography was performed in patient 1 and signs of acute denervation of the right flexor digitorum profundus were found (Figure 1E). Cerebrospinal fluid (CSF) analysis excluded the presence of malignant cells. Serum complement C3 and C4 levels were in the normal range, and serological markers for systemic vasculitis (antinuclear antibodies, ANA; extractable nuclear antigen antibodies, ENA; antineutrophil cytoplasmic antibodies, ANCA; cryoglobulins) were negative in all patients, except for ANA (grainy speckled pattern, titer 1:640), detected in patient 3 with anti-Ro/SSA positivity (titer 1:480); as this patient had no symptom of Sjögren’s disease, lip biopsy for minor salivary gland was not performed. Onconeural antibodies were negative in all patients. Peripheral nerve biopsy found axonal degeneration in all patients. Necrotizing vasculitis on nerve biopsy was observed in patients 1 and 3; in patient 2, marked IgA deposition was detected without inflammatory lesions or amyloid deposits (Figures 2A–C). Histological evidence of vasculitis was also observed in the skin biopsy of a purpuric lesion of patient 1 and fibularis brevis muscle biopsy of patient 3 (Figure 2D). ICIs were permanently withdrawn, and corticosteroids were administered to all patients. Response to corticosteroids varied: Patient 1 experienced transient improvement (CTCAE grade 2), but at tapering of corticosteroids, symptoms recurred, spreading to left radial and ulnar nerve territories (CTCAE grade 3). Symptoms then stabilized again following plasma exchanges and intravenous corticosteroids. In patient 2, neurological symptoms (and arthralgia) completely regressed (CTCAE grade 0); patient 3 experienced slight motor improvement but no substantial change of disability (CTCAE grade 3). Second-line treatments (cyclophosphamide and rituximab) were administered to patient 1 only; although 1 month later, symptoms had extended to the left tibial, left sural, and bilateral fibular nerves, the patient started to improve 3 months after second-line treatment (CTCAE grade 2, Table 1). The last nerve conduction studies performed (2–6 months from onset) demonstrated stability (patient 2) or a slight increase (patient 1 and patient 3) of the amplitudes of motor and sensory action potentials of the affected nerves (Supplementary Table S1). At last visit (median 8 months, range 5–10), all patients were stable (CTCAE grade 0–3). Cancer progression was documented in patient 1 and patient 3 (Table 1).

**Table 1:** Data of individual patients.

	Patient 1	Patient 2	Patient 3
Age (years), sex	72, male	88, male	86, male
Previous medical history	Unremarkable	Lumbar canal stenosis, psoriatic arthritis treated with low-dose prednisone (5 mg/day)	L5-S1 disk herniation in 1970 without neurological sequelae; weaned alcohol abuse
Mesothelioma histological subtype	Epithelioid	Sarcomatoid	Epithelioid
Number of ICI cycles	10	4	6
Neurological examination	Weakness (abductor pollicis brevis and finger flexor at 3/5 on the MRC scale, and index finger flexor at 1/5 on the MRC scale), sensory loss, allodynia, and areflexia (loss of brachioradialis reflex) of bilateral hand (median nerve territory)	Weakness (right anterior tibial 2/5 on the MRC scale, left anterior tibial, bilateral quadriceps, and gastrocnemius 3/5 on the MRC scale) and areflexia of lower limbs, four-limb allodynia, and sensory ataxia	Weakness (left anterior tibial, bilateral interosseous, and left extensor digitorum communis 0/5 on the MRC scale), sensory loss, allodynia of left foot and bilateral hand (left radial and ulnar territory, right ulnar territory), areflexia, bilateral interosseous amyotrophy, and sensory ataxia
Nerve conduction study; electromyography	Axonal sensory motor neuropathy of median nerves; lack of voluntary muscle contraction and active denervation in left and right abductor pollicis brevis and right flexor carpi radialis	Lower limb asymmetric axonal sensory motor neuropathy: active denervation in left anterior tibial, left peroneus longus, left vastus lateralis, right medial gastrocnemius, right vastus medialis, and left vastus lateralis	Four-limb axonal sensory motor neuropathy (predominantly affected nerves: left median, left ulnar, left radial, and left fibular); lack of voluntary muscle contraction and active denervation in left and right dorsal interosseous, left extensor digitorum communis, and left anterior tibial
Cerebrospinal fluid	0 cells/mm^3^, proteins 0.34 g/L, IL-6 2.1 pg/mL, no oligoclonal bands	0 cells/mm^3^, proteins 0.33 g/L, IL-6 2.6 pg/mL, no oligoclonal bands	1 cell/mm^3^, proteins 0.63 g/L
C reactive protein, mg/L	18.4	213.5	127.1^*^
Autoantibodies	ANA, ENA, ANCA, onconeural^**^ negative	ANA, ENA, ANCA, onconeural^**^ negative	ANA 1/640, Ro/SSA 1/480; ANCA, onconeural^**^ negative
Non-neurological symptoms	Purpuric lesions on hands and feet	Scapular and pelvic girdles arthralgias	Absent
CTCAE grade	3	3	3
Corticosteroids	IVMP (120 mg daily for 3 days, 240 mg daily for another 3 days), then oral PDN 1 mg/kg daily	Oral PDN 240 mg daily for 3 days, then 1 mg/kg daily	Intravenous prednisolone 7.5 mg/kg daily for 3 days, then oral PDN 1 mg/kg daily
Response to corticosteroids	Transient improvement	Full recovery	Slight improvement
Disease course after first-line therapy	Stepwise progression: symptoms recurred and spread to the left ulnar and radial nerve; then, to left tibial, left sural, and left and right fibular nerves	Non-progressive	Non-progressive
Second-line treatment	Cyclophosphamide (1,000 mg monthly, 5 cycles) and rituximab (1,000 mg 14 days apart, 1 cycle)	None	None
Tapering of corticosteroids	3 months after onset, reduction of the daily oral PDN dose of 5 mg/week	1 month after onset, reduction of the daily oral PDN dose of 5 mg/week	1 month after onset, reduction of the daily oral PDN dose of 10 mg/2 weeks
Cancer progression (months from onset)	Yes (4)	No	Yes (4)
Cancer treatment after immune checkpoint inhibitor withdrawal	Carboplatin and pemetrexed	No	Carboplatin and pemetrexed
Follow-up duration (months)	10	8	5
CTCAE grade last visit	2	0	3

^*^Concomitant sepsis (cutaneous and peripheral venous catheter infection). ^**^Hu, Yo, Ri, Cv2, amphiphysin, Ma2, SOX1, GAD65, Tr, Zic4, and titine. ANA, Antinuclear antibodies; ANCA, Antineutrophil cytoplasmic antibodies; CTCAE, Common Terminology Criteria for Adverse Events; GAD, Glutamic acid decarboxylase; ICI, Immune checkpoint inhibitor; IL-6, Interleukin 6; IVMP, Intravenous methylprednisolone; MRC, Medical Research Council; PDN, Prednisone; and SSA, Sjögren’s syndrome-related antigen A autoantibodies.

## 4 Discussion

Three cases presented herein had a mononeuritis multiplex leading to suspect the diagnosis of vasculitic neuropathy (5). Sural biopsy demonstrated vasculitis (definite diagnosis) (5) in patients 1 and 3, while only axonal degeneration and IgA deposits were found in patient 2. Although nerve biopsy is negative in 40–50% of patients with a high level of clinical suspicion for vasculitic neuropathy (5), the mechanism underlying mononeuritis multiplex in patient 2 may be different from nerve vasculitis as suggested by a more rapid and better response to corticosteroids. In the other two patients, both with histologically proven vasculitis, response to corticosteroids was incomplete or transient, indicating that immunosuppressive treatments such as cyclophosphamide and rituximab, which led to clinical improvement in patient 1, might be necessary as recommended by the Peripheral Nerve Society guideline outside the ICI context (5).

After a decade of extensive use of ICIs, only a handful of definite or probable cases of vasculitic neuropathy have been described (Supplementary Table S2) (6–11), and they represent less than 1% of all reported n-irAEs (3), a rarity confirmed in our database, as patients with mononeuritis multiplex were 3/183 (1.7%) of patients with CTCAE grade ≥ 2 n-irAEs identified in more than 7 years. ICIs were approved only recently (in France, June 2021) in unresectable malignant mesothelioma (4). Earlier, vasculitic neuropathy was reported in two mesothelioma patients treated off-label with anti-PD-1 monotherapy (10, 11), and a retrospective pharmacovigilance study identified 10 cases of neuropathy and two of vasculitis among 23 patients with mesothelioma experiencing n-irAEs (12). In addition, there is one recently published report of cutaneous vasculitis, reminiscent of patient 1, in a mesothelioma patient treated with combined nivolumab and ipilimumab (13). Herein, definite or possible vasculitic neuropathy was observed in nearly half (3/7) of mesothelioma patients included in a large cohort of patients with n-irAEs, and we did not observe it in any other cancer type, leading to the speculation of a possible association between mesothelioma, ICIs, and vasculitic neuropathy, that future studies need to confirm.

The mechanism driving the inflammatory process, however, remains unknown. The relevance of anti-SSA/Ro antibodies without signs indicative of Sjögren’s syndrome in patient 3 is uncertain, although diagnostic tests to exclude a subclinical *sicca syndrome* such as chewing gum test, Schirmer’s test, sonography of salivary glands, or lip salivary biopsy were not performed. Similarly, the significance of IgA deposits in the nerve tissue of patient 2 is unclear. Even if antineutrophil cytoplasmic antibodies were reported in some melanoma patients who developed vasculitic neuropathy following ICI treatment (6, 8), suggesting similarities with primary systemic vasculitides (5), they tested negative in all mesothelioma patients with vasculitic neuropathy, whether presented herein or elsewhere (10, 11), which suggests distinct mechanisms. Possibly, a two-step process is involved, in which mesothelioma cells first prime the immune system, with the driving effect of ICI treatment needed for the disease to clinically develop, as previously suggested for other n-irAEs (14). In support of this hypothesis, paraneoplastic vasculitis was reported in some mesothelioma patients without ICI treatment (15). Further research is needed to assess whether autoantibody positivity at baseline could predict the development of mononeuritis multiplex in patients receiving ICIs.

To conclude, clinicians must be aware that mononeuritis multiplex, definitively or possibly due to vasculitis, may occur in patients treated with ICIs and is treatable. The occurrence of this very rare adverse event of ICIs in three patients with mesothelioma might suggest a possible association between mononeuritis multiplex, ICIs, and mesothelioma, but other studies are needed to confirm this speculation.

## Data availability statement

The original contributions presented in the study are included in the article/Supplementary material, further inquiries can be directed to the corresponding author.

## Ethics statement

The studies involving humans were approved by the Institutional Review Board of the Université Claude Bernard Lyon 1 and Hospices Civils de Lyon (69HCL21-474). The studies were conducted in accordance with the local legislation and institutional requirements. The participants provided their written informed consent to participate in this study. Written informed consent was obtained from the individual(s) for the publication of any potentially identifiable images or data included in this article.

## Author contributions

AF: Conceptualization, Data curation, Formal analysis, Investigation, Methodology, Writing – original draft, Writing – review & editing. ME: Conceptualization, Data curation, Formal analysis, Investigation, Methodology, Writing – original draft, Writing – review & editing. MD: Writing – review & editing. MM: Writing – review & editing. AP: Writing – review & editing. MV-G: Writing – review & editing. PD: Writing – review & editing. AL: Writing – review & editing. AS: Writing – review & editing. KA: Writing – review & editing. AW: Writing – review & editing. LG: Writing – review & editing. NS: Writing – review & editing. TM: Writing – review & editing. JH: Funding acquisition, Writing – review & editing. CB: Writing – review & editing. DP: Writing – review & editing. DW-L: Conceptualization, Data curation, Methodology, Supervision, Writing – review & editing. BJ: Conceptualization, Data curation, Funding acquisition, Methodology, Supervision, Writing – review & editing.
