# Supplementary material for: Mononeuritis multiplex following immune checkpoint inhibitors in malignant pleural mesothelioma
Source: Front Neurol. 2024 Jan 25;15:1338899. doi: 10.3389/fneur.2024.1338899 (PMC10850347; doi:10.3389/fneur.2024.1338899)
Supplement: Supplementary file 1 [file Table_1.DOCX]

Supplementary Material

# Supplementary Tables

**Table S1. Nerve amplitudes on nerve conduction studies.**

|  |  |  |  | **Patient 1** | | | | **Patient 2** | | | **Patient 3** | |
| --- | --- | --- | --- | --- | --- | --- | --- | --- | --- | --- | --- | --- |
| **Nerve** | **Type** | **Record site (side)** | **Normal amplitude** | **Day 28** | **Day 36** | **Day 84** | **Day 208** | **Day 20** | **Day 74** | **Day 253** | **Day 21** | **Day 49** |
| Med | Motor | APB (right) | > 4 | ***NR*** | ***NR*** | ***NR*** | ***NR*** | 5.2 | - | - | ***3.5*** | 4.7 |
| Med | Motor | APB  (left) | > 4 | ***0.1*** | ***NR*** | ***NR*** | ***0.3*** | 5.6 | 4.5 | 5.5 | ***NR*** | ***0.36*** |
| Uln | Motor | ADM (right) | > 4 | 6.8 | 8.3 | 7.3 | 7.8 | 5.0 | - | - | ***1.0*** | ***1.0*** |
| Uln | Motor | ADM (left) | > 4 | 6.1 | 5.6 | ***3*** | 4.9 | 5.1 | 4.9 | 4.7 | ***NR*** | ***NR*** |
| Rad | Motor | EIP (right) | > 2.5 | - | 4.1 | 4 | 3.1 |  | - | - | 3.8 | - |
| Rad | Motor | EIP (left) | > 2.5 | 3.5 | ***2.4*** | ***0.5*** | ***1.5*** |  | 4.4 | 3.4 | ***NR*** | ***0.4*** |
| Fib | Motor | EDB (right) | > 2 | 2.1 | - | ***0.6*** | ***0.8*** | ***NR*** | ***-*** | ***-*** | ***NR*** | ***NR*** |
| Fib | Motor | EDB (left) | > 2 | 2.1 | - | ***0.6*** | ***0.8*** | ***0.1*** | ***NR*** | ***NR*** | ***NR*** | ***NR*** |
| Fib | Motor | Tib Ant (right) | > 3 | ***2.0*** | - | ***1.4*** | ***2.7*** | ***1.2*** | - | ***1.0*** | ***2.1*** | ***2.2*** |
| Fib | Motor | Tib Ant (left) | > 3 | - | - | 3.9 | 4.5 | ***2.8*** | ***1.2*** | ***-*** | ***0.1*** | ***0.1*** |
| Tib | Motor | AHB (right) | > 4 | ***2*** | - | ***1.5*** | ***2.5*** | ***NR*** | - | - | - | ***0.14*** |
| Tib | Motor | AHB (left) | > 4 | 6.3 | - | ***3.2*** | ***3.3*** | ***1.4*** | ***1.4*** | ***0.9*** | ***NR*** | ***NR*** |
| Med | Sensory | D3 (right) | > 20 | ***NR*** | - | - | ***NR*** | ***NR*** | - | - | ***NR*** | ***1.88*** |
| Med | Sensory | D3 (left) | > 20 | ***NR*** | ***NR*** | - | ***NR*** | ***NR*** | ***4.7*** | ***3.6*** | ***NR*** | - |
| Uln | Sensory | D5 (right) | > 20 | 20 | 30 | - | ***15*** | ***2.7*** | - | - | ***NR*** | ***NR*** |
| Uln | Sensory | D5 (left) | > 20 | ***13*** | ***11*** | - | ***7*** | ***8.1*** | ***8.5*** | ***6.6*** | ***NR*** | - |
| Rad | Sensory | (right) | > 18 | 34 | 35 | 42 | 35 | - | - |  | ***9.1*** | ***7.4*** |
| Rad | Sensory | (left) | > 18 | 41 | 37 | ***NR*** | ***NR*** | 10.4 | 14.7 | 13.4 | ***NR*** | - |
| Sur | Sensory | (right) | > 10 | ***5*** | ***7*** | ***NR*** | ***NR*** | ***NR*** | - | - | ***NR*** | ***NR*** |
| Sur | Sensory | (left) | > 10 | ***8*** | 12 | ***NR*** | ***3*** | ***NR*** | ***NR*** | - | ***NR*** | - |
| Sup Fib | Sensory | (right) | > 8 | 10 | 10 | ***3*** | ***3*** | - | - | - | ***NR*** | - |
| Sup Fib | Sensory | (left) | > 8 | ***6*** | ***6*** | ***NR*** | ***NR*** | - | - | - | ***NR*** | - |

The table shows the amplitudes of the compound motor action potentials (mV) and sensory action potentials (µV) of the explored nerves in the 3 patients at onset and during follow-up. Sensory action potential amplitudes were measured using an antidromic technique. In bold and italic, the abnormal value of amplitude.

Abbreviations. *ADM* Abductor digiti minimi, *AHB* Abductor hallucis brevis, *APB* Abductor pollicis brevis, *Ant* Anterior, *D* Digit, *EDB* Extensor digitorum brevis, *EIP* Extensor indicis proprius, *Fib* Fibular, *L* Left, *Med* median, *NR* Not recorded, *R* Right, *Rad* Radial, *Uln* Ulnar, *Sup* Superficial, *Sur* Sural, *Tib* Tibial.

**Table S2. Previously published patients with immune checkpoint inhibitor-related mononeuritis multiples/peripheral nerve vasculitis**

| **Reference** | **Sex, age (years)** | **Cancer** | **ICI (number of doses)** | **Clinical presentation** | **NCS/EMG** | **Nerve biopsy** | **Serum screening for systemic vasculitis** | **Involvement of other organs** | **Treatment** | **Outcome** |
| --- | --- | --- | --- | --- | --- | --- | --- | --- | --- | --- |
| Villarreal-Compagny et al.^6^ | F, 37 | Melanoma | Ipilimumab (3) | Generalized itchy and weakness with sensory loss of right LL. | Mononeuritis multiplex | Vasculitis of the vasa nervorum | c-ANCA and p-ANCA positive | No | PDN, MMF | Complete recovery |
| Sakai et al.^7^ | M, 81 | Melanoma | Nivolumab (1) | Symmetric LL proximal muscles weakness followed by left hand weakness and left foot drop. Neurological examination showed left ulnar nerve and bilateral peroneal nerve palsies. | Mononeuritis multiplex | Not performed | Negative | Skin (livedo reticularis), muscle (rhabdomyolysis) | IVMP, PDN | Partial improvement |
| Dubey et al.^8^ | M, 77 | Melanoma | Pembrolizumab (1) | Painful, sequential foot drop followed by  bilateral hand numbness and weakness and sensory ataxia. Neurological examination demonstrated weakness and sensory loss of individual peripheral nerves (median, ulnar, radial,  peroneal). | Mononeuritis multiplex | Not performed | p-ANCA positive, ↑ESR | No | IVMP, PDN, rituximab | Partial improvement |
| Abdelhakim et al.^9^ | F, 80 | Melanoma | Ipilimumab and nivolumab (3) | Left LL weakness followed by left foot drop, weakness of left hand. Neurological examination showed symmetric muscle weakness (2/5-4/5 on MRC scale), left calf atrophy | Mononeuritis multiplex | Endoneural fibrosis and scattered endoneural macrophages without vasculitis | ANA positive (1:160) | No | IVIG, IVMP, PDN, rituximab | No improvement |
| Kao et al.^10^ | M, NA | Mesothelioma | Nivolumab (5) | Asymmetric distal ULs limbs and LLs sensory loss and weakness | Severe length-dependent axonal sensorimotor neuropathy | Necrotizing vasculitis | ↑ESR | No | IVMP, PDN | Partial improvement |
| Baldauf et al.^11^ | F, 61 | Mesothelioma | Pembrolizumab (25) | Bilateral foot drop and paresis in the distribution of the right ulnar nerve, hypoesthesia/allodynia of the feet, the right hand, and the left thumb. | Mononeuritis multiplex | Small vessel vasculitis | Negative | Skin (petechiae, histologic demonstration of vasculitis) | IVMP, PND, CYC | No improvement |

Abbreviations. *ANA* antinuclear antibodies, *ANCA* antineutrophil cytoplasmic antibodies, *CYC* cyclophosphamide, *EMG* electromyography, *ESR* erythrocyte sedimentation rate, *F* female, *ICI* immune checkpoint inhibitor, *IVIG* intravenous immunoglobulin, *IVMP* intravenous methylprednisolone, *LL* lower limb, *M* male, *MMF* mycophenolate mofetil, *MRC* Medical Research Council, *NCS* nerve conduction studies, *PDN* prednisone, *UL* upper limb.
